# Advances in the Application and Mechanism of Admixtures and Industrial By-Products in Cement-Based Self-Leveling Mortar: A Comprehensive Review

**DOI:** 10.3390/ma18081709

**Published:** 2025-04-09

**Authors:** Meirong Zong, Haozhe Ma, Xiancui Yan, Pinghua Zhu, Wenhao Wang, Hui Liu, Faqin Dong, Minqi Hua

**Affiliations:** 1 Department of Civil Engineering, Changzhou University, Changzhou 213164, China; 13525413493@163.com (H.M.); yanxc@cczu.edu.cn (X.Y.); zph@cczu.edu.cn (P.Z.); 19501141529@163.com (W.W.); liuhui@cczu.edu.cn (H.L.); 2 Key Laboratory of Solid Waste Treatment and Resource Utilization, Ministry of Education, Southwest University of Science and Technology, Mianyang 621010, China; fqdong@swust.edu.cn; 3 School of Civil Engineering & Architecture, Wuhan University of Technology, Wuhan 430070, China; hmq@whut.edu.cn

**Keywords:** cement, self-leveling mortar, admixture, industrial by-product

## Abstract

Cement-based self-leveling mortar (CSL) is a special building material that utilizes cement as the main cementitious component, combined with a variety of admixtures. Its self-leveling characteristics enable it to effectively level and fill uneven surfaces. Additionally, the innovative green CSL developed from industrial by-products can further enhance both environmental and economic benefits. This paper systematically reviews the use of admixtures and industrial by-products in the production of CSL. The main findings include the following: (i) compared to the international ISO standard, China’s standard JC/T 985 provides more detailed testing parameters regarding fluidity, mechanical properties, and shrinkage; (ii) the effect of additives on CSL is affected by its molecular weight and structure, and high molecular weight improving the workability of the additives; (iii) industrial by-products have been effectively incorporated into CSL, leading to a reduction in reduced greenhouse gas emissions and a decreased environmental impact; (iv) macro and microanalysis results of different green CSLs show that industrial by-product gypsum has the greatest potential for application in CSL. Based on these findings, this paper offers valuable reference data for the use of admixtures and industrial by-products in CSL. Furthermore, it contributes innovatively to the sustainable development of infrastructure construction.

## 1. Introduction

According to the definition provided by the China Cement Products Standardization Technical Committee, cement-based self-leveling mortar (CSL) is a specialized mortar composed of cement-based binding materials, aggregates, fillers, and admixtures, which exhibits high fluidity when mixed with water [1]. Due to its low cost, simplified construction process, rapid curing time, and smooth surface finish, CSL has been widely utilized in various applications, including flooring materials, indoor flooring, building walls, and 3D printing [2,3,4,5,6,7,8,9]. In its flowing state, CSL possesses excellent fluidity, pumpability, and self-leveling characteristics, which significantly streamline the construction process and minimize the need for manual intervention [10]. Furthermore, CSL demonstrates rapid setting properties, achieving immediate load-bearing capacity that is suitable for high-traffic and high-pressure applications shortly after curing [11].

In order to address potential issues such as delamination, segregation, and shrinkage cracking in CSL, a variety of admixtures are typically incorporated during the design phase of the mixture to enhance its performance. These admixtures include water reducers, water retainers, set retarders, defoamers, and expansion agents, among others [12,13]. Ge et al. [14] studied the effects of four water reducers: sodium lignosulfonate (LS), naphthalene superplasticizer (PNS), aliphatic superplasticizer (HSB), and polycarboxylate superplasticizers (PCE) on the fluidity of a cement slurry. They found that PCE provides the best dispersion performance for cement while achieving high fluidity and reducing the volume shrinkage of CSL. Patural et al. [15] conducted a comparative analysis of cellulose ether water-retaining agents with varying molecular weights, assessing their effects on the water retention and consistency of cement-based mortar. Their results demonstrated that an increase in the molecular weight of the water-retaining agent corresponded with improved consistency and water retention in the mortar. Wang et al. [13] evaluated the influence of different dosages of cellulose ether water-retaining agent (CE), tartaric acid retarder (TA), and PCE on CSL performance, identifying optimal dosages of CE at 0.6 wt‰, TA at 0.5 wt‰, and PCE at 2.0 wt‰. The incorporation of these admixtures can lead to a reduction in both the water–cement ratio and the cement ratio, while simultaneously enhancing the fluidity, surface characteristics, and mechanical properties of the mortar. Nevertheless, it is important to note that while the addition of various admixtures enhances the durability of CSL, it also results in increased economic costs [16,17]. Pan et al. [18] used carrot extract as a retarder for cement mortar; this natural admixture not only acts as a setting retarder, but also enhances compressive strength. The use of natural admixtures can meet the performance requirements of CSL while providing significant environmental and economic benefits.

Traditional CSL relies on Portland cement (PC) as the main cementitious material, and the CO_2_ emissions generated by its production process account for 8% of global CO_2_ emissions and consume significant natural resources. Meanwhile, the annual emission of industrial by-products exceeds 4 billion tons, with long-term accumulation leading to environmental issues such as land occupation and heavy metal pollution [5,19]. Although the performance of CSL can be optimized by adding additives, existing research still faces bottlenecks such as environmental and cost conflicts, and insufficient utilization of solid waste [20,21,22]. Lin et al. [5] found that phosphogypsum (PG) in CSL not only serves as a filler, but also directly participates in the hydration process of cement in self-leveling mortar. Wang et al. [23] found that incorporating TG into CSL enhances the density of the mortar’s structure, thereby improving both its strength and deformation capacity. Additionally, CSL mixed with industrial by-product gypsum offers superior thermal insulation, sound insulation, fire resistance, and environmental comfort, while also reducing carbon emissions by 20% [24]. Ceramic waste is a non-degradable material that can take up to 4000 years to decompose and is challenging to recycle [25]. Gruber et al. [26] investigated the use of PG and white ceramic waste to replace 50% of PC in CSL. The compressive strength of the CSL containing 50% white ceramic waste was measured at 32.66 ± 4.26 MPa after 28 days, demonstrating very low shrinkage and hydration heat. The mixed mortar with 50% PG also exhibited strong compressive, flexural, and bonding strengths. Given the low biodegradability of plastic waste and its substantial volume, processing this waste presents significant benefits for environmental protection. Safi et al. [27] investigated the use of recycled waste plastics as fine aggregate in CSL. The study found that increasing the plastic waste content enhanced the fluidity of the mortar. Although the compressive strength of mortar containing 20–50% plastic waste decreased by 15% to 33%, it still meets the mechanical performance requirements for CSL, as specified in the Chinese standard JC/T 985-2017 [1].

Due to the differences in test methods between international (ISO) and Chinese standard (JC/T 985-2017), high-performance admixtures are expensive, and excessive use may delay the development of strength; the dosage threshold of industrial by-products to replace cement, the pretreatment process, and its influence on the hydration mechanism are still unclear. This paper first analyzes the differences in the requirements for CSL fluidity, mechanical properties, and durability between Chinese and Western standards, establishing a universal performance evaluation framework. Secondly, it reveals the regulatory mechanisms of admixture molecular structure and molecular weight on the rheological properties and hydration process of CSL. Then, the key performance parameters of CSL produced by replacing cement with different industrial by-products are provided. It quantifies the feasibility of substituting cement with PG (≤55%), TG (≤45%), ceramic waste (50%), etc., and elucidates the mechanisms of their participation in hydration reactions. Based on the collection of existing research, a summary table of basic research information on the preparation of CSL from different industrial by-products is given. Finally, several constructive suggestions are put forward for future research on the use of admixtures and industrial by-products in CSL. This review provides help for the application and development of CSL.

## 2. CSL Test Methods and Standards

The physical properties of CSL in a flowing state primarily include fluidity and setting time. Fluidity is a critical factor that influences the ability of CSL to self-level the floor substrate. ISO 9597:2008 [28] provides stringent guidelines for the fluidity testing method. First, the mixed mortar is poured immediately into an open pipe mold with a diameter of 40 mm and a height of 50 mm. Next, the mold is lifted vertically by 50–100 mm within 2 s and held for 10–15 s to allow for the mortar to flow freely onto a glass plate. After 4 min, the diameter of the mortar is measured in two perpendicular directions; this measurement represents the initial fluidity. The diameter of the CSL is also measured after 20 min, referred to as the 20 min flowability. According to the Chinese Standard JC/T 985-2017 [1], the fluidity of qualified CSL should be ≥130 mm. The setting time directly influences the mortar’s ability to flow freely during application, the degree of void filling, and the smoothness of the floor surface. ISO 9597:2008 [28] specifies that the initial and final setting times of mortar should be measured using a Vicat needle apparatus. The initial setting time is defined as the duration from the completion of mixing until the Vicat needle penetrates less than 5 mm, while the final setting time is determined when the Vicat needle penetrates less than 1 mm. An excessively short setting time may lead to premature solidification of the mortar, adversely affecting its final strength and durability.

Mechanical properties such as tensile bond strength, impact resistance, abrasion resistance, and dimensional change rate are critical characteristics of CSL in their hardened state. These properties are influenced by the components of the mixture and their respective proportions. Typically, mortar prisms measuring 40 mm × 40 mm × 160 mm are prepared, demolded after curing for 24 h, and subjected to performance testing after a specified period. Compressive strength and flexural strength tests are conducted at day 1 day and day 28 in accordance with ISO 679:2009 [29] for subsequent mechanical evaluation. A high tensile bond strength ensures that the CSL adheres securely to the underlying surface, contributing to the formation of a smooth finish. ISO 13007-2:2013 [30] stipulates that the specimen cured for 27 days should be bonded to the instrument. After 1 day, the load should be applied at a speed of (5 ± 1) mm/min until the specimen is destroyed. The tensile bond strength is calculated according to Formula (1). JC/T 985-2017 [1] requires the CSL test block not to crack or detach from the base plate after the impact resistance test. ISO 10545-6:2010 [31] recommends using corundum with a particle size of F80 as an abrasive to test the wear resistance of CSL at 75 r/min, and believes that after 150 revolutions, the CSL test block with a grinding pit volume less than 400 mm^3^ has better wear resistance. According to the Chinese standard JGJ/T 70-2009 [32], the drying shrinkage test of CSL specimens was carried out. First, the reference length of each group was measured after the specimens were cured for 24 h. Then, the length of each specimen after a certain number of days of curing was recorded, and its drying shrinkage rate was determined according to Formula (2). Table 1 summarizes the characteristics of CSL, and the standards and requirements based on the specifications.

(1) $$P=\frac{F}{S}$$

where P is the tensile bond strength (MPa); F is the maximum failure load in Newtons (N); and S is the bonding area (mm^2^).

(2) $$\varepsilon =\frac{L_{0}-L_{n}}{L-L_{d}}\;\times \;100\%$$

where ε is the shrinkage value, L_0_ is the initial length of the sample, L_n_ is the length after n days, L = 160 mm, L_d_ is the sum of the lengths of the two shrinkage heads buried in the mortar sample.

## 3. CSL Component Material Types and Characteristics

### 3.1. Cementitious Materials

In recent decades, rising labor costs and increasing user demand for mortar workability (rapid setting, fluidity, shrinkage compensation, and smoothness) have led to the widespread use of CSLs with high fluidity and self-smoothing properties. These materials are now commonly employed in various sectors, including office buildings, apartments, retail spaces, schools, hospitals, factories, parking lots, ships, and other new construction and maintenance projects [4,11,33,34,35]. As mentioned above, cementitious materials, aggregates, fillers, admixtures, and water typically compose CSL systems [5,20,24,36,37]. PC is the most commonly used binder in CSL, as it enhances the material’s cohesion and mechanical properties [38]. However, the production of PC is invariably associated with significant CO_2_ emissions and high energy consumption, which has become a pressing resource and environmental concern in the application of CSL [16]. Calcium sulfoaluminate cement (CSA) is regarded as a more environmentally friendly and cost-effective binder for self-leveling systems. Compared to PC, CSA clinker requires less limestone in the manufacturing process, has a lower calcination temperature, and exhibits greater brittleness. These characteristics contribute to reduced carbon dioxide emissions and lower energy consumption [39,40,41,42,43]. Li et al. [16] utilized CSA to replace PC in a traditional ternary system consisting of PC, calcium aluminate cement (CAC), and calcium sulfate, resulting in the preparation of CSL with commendable performance. The hydration of CAC-based mortar is mainly related to the formation of ettringite and aluminum hydroxide. When the calcium sulfate in the system is depleted, ettringite dissolves, and monosulfate precipitates [4]. From a hydration perspective, calcium sulfoaluminate (CSA) also produces ettringite and aluminum hydroxide as hydration products. Conversely, once the sulfate in the pore solution is depleted and sufficient free water is available, monosulfate is formed. Additionally, the cost of CSA is only 25–50% of that of CAC, and CSL prepared with CAS cement clinker exhibits excellent mechanical properties and shrinkage compensation [11].

### 3.2. Aggregate

Sand is typically the aggregate and main component of a CSL mixture. The type, particle size, and shape of the sand significantly influence the fluidity, consistency, strength, water consumption, and segregation of CSL, as well as the production cost. In a study by Scolaro and Rocha [36], the effects of three different types of quartz sand on the performance of CSL were examined. The findings indicated that the shape and texture of the sand particles did not appear to have a substantial impact on the performance of CSL. The finer the particle size of sand, the better the performance of CSL. He et al. [44] proposed morphological indexes for sand particles with varying gradations. Their study concluded that the performance of sand particles exhibiting mortar morphology with influence factors between 0.86 and 1.08 is relatively excellent. Zhang et al. [45] utilized low-grade fly ash as a substitute for natural river sand in the preparation of mortar, which demonstrated good performance. This approach significantly contributes to alleviating the shortage of natural river sand resources, reducing pollution, and enhancing the utilization of solid waste resources. Canbaz et al. [46] produced CSL using three types of aggregates and a new generation of high-efficiency water reducers. They conducted a series of tests to evaluate slump, compressive strength, weight, ultrasonic pulse velocity, water absorption, shear strength, and other properties. The results indicated that finer aggregate particle sizes improved material fluidity. Notably, river sand with a particle size of 0–1 mm combined with 1% water reducer exhibited the best workability in CSL.

### 3.3. Filling Material

With the rapid development of industry and urbanization, the amount of solid waste generated in cities is increasing. Solid wastes such as fly ash (FA), limestone powder (LSP), waste brick powder (WBP), steel slag, and desulfurization ash have significantly impacted ecological development, and their treatment and utilization have consistently attracted considerable attention [47,48,49,50]. In recent years, the incorporation and utilization of these solid wastes in CSL have gradually become a prominent topic in both research and application. FA, which has a high content of silicon and alumina, features spherical particles that are similar in size to ordinary cement, making it an excellent additive for CSL [51]. Previous studies have demonstrated that FA can enhance the flowability and stability of mortar, as well as improve the densification and mechanical properties of its structure [2,52,53]. Moreover, FA can slow the setting time of mortar, facilitate pouring, and decrease early heat generation, thereby minimizing shrinkage. LSP filler is another commonly used material that can increase the filling capacity of the cement particle skeleton while maintaining the cohesiveness and anti-segregation properties of cement-based systems [54,55]. Reusing industrial waste and by-products as fillers for CSL raw materials is another advantage and can also be considered a sustainable waste solution [38]. The ceramic brick industry produces a significant amount of sintered clay brick waste, which typically exhibits some pozzolanic activity and can react with calcium hydroxide to form compounds that enhance strength and durability [56,57]. Zhao et al. [58] utilized WBP to partially or fully replace limestone filler at rates of 50% and 100%, resulting in early strength reductions of 5.6% and 9.3% in the mortar, respectively. However, this decline was mitigated by the pozzolanic activity of WBP, which further improved the strength at 28 days. Rizwan et al. [59] investigated the effects of mixed materials derived from various solid wastes on the fluidity, strength, microstructure, relative water absorption, and early volume stability of cement-based systems. They found that the inclusion of FA and LSP in the solid waste resulted in improved overall performance of the mortar. Xu et al. [60] discovered that incorporating a small amount of LSP to replace FA in mortar can enhance the strength of CSL. LSP effectively fills the pores of the mortar and promotes the formation of AFt. However, as the content of LSP increases, some LPs that do not participate in the reaction may have a dilution effect, leading to a reduction in the mechanical properties of the mortar. These studies provide valuable data and support for the application of limestone powder and fly ash composite systems, as well as various solid wastes in CSL.

### 3.4. Types of Admixtures in CSL

#### 3.4.1. Water Reducing Agent

**Figure 1:**
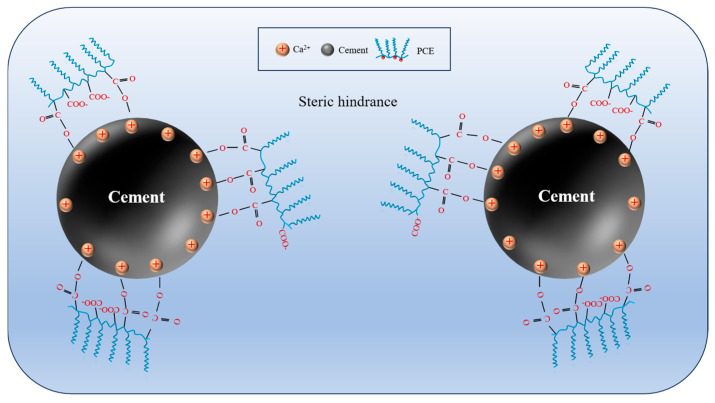
Schematic diagram of the PCE adsorption process on the cement particle surface.

High-efficiency water reducers have become essential CSL formulations. Over the past 30 years, polycarboxylate superplasticizers (PCEs) have emerged as the most widely used superplasticizers in CSL due to their high water reduction rates and the ease with which their polymer structures can be modified to achieve desired properties [13,37,61,62]. As illustrated in Figure 1, when PCE is introduced into the cement slurry system, it is rapidly adsorbed onto the surfaces of cement particles due to the strong electrostatic interactions between the carboxylic acid groups in PCE and the highly charged mineral surfaces [63,64]. Concurrently, the steric effects between cement particles result in a significant increase in their dispersibility, thereby facilitating a high water reduction rate [65].

The effects of water reducers on the CSL fluidity, setting time, and crystal morphology of cementitious materials depend on the length and density of the side chain groups. Dalas et al. [66] analyzed the influence of PCE side chain length, density, and anion functionality (carboxylate, dicarboxylate, or phosphate) on adsorption. They found that modifying the anion is an effective technical approach to enhance PCE performance. Chuang et al. [67] observed that as the length of the PCE side chain group increased, the steric hindrance effect in the mortar became more pronounced, leading to greater dispersion among the cement molecules. Specifically, longer side chain groups generate greater steric hindrance around themselves. Furthermore, Liu et al. [68] discovered that PCE adsorption reduces the hydration rate of the mortar and alters the morphology and size of the crystals. This delayed hydration caused by comb PCE can also impede the early strength development of the mortar, negatively impacting its overall strength growth [62].

**Figure 2:**
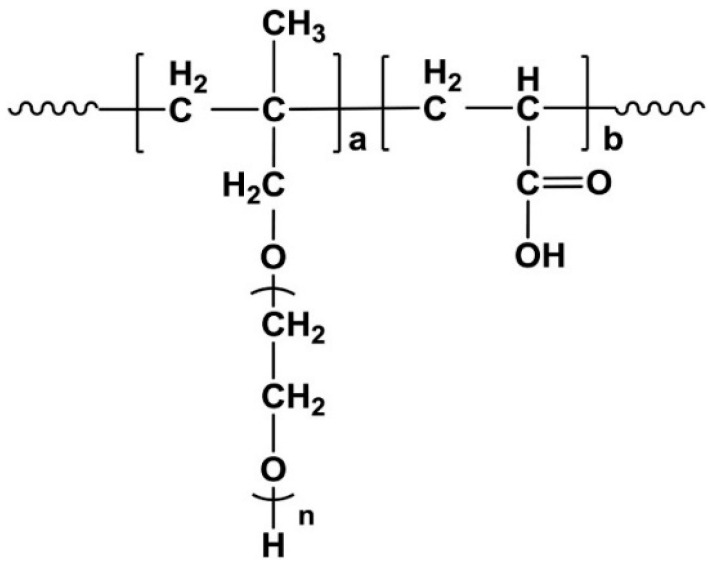
Chemical formula for synthesizing PCEs (n = 89, 53, 10; a:b = 1:2, 1:4, 1:6) [62] (permission has been granted).

**Figure 3:**
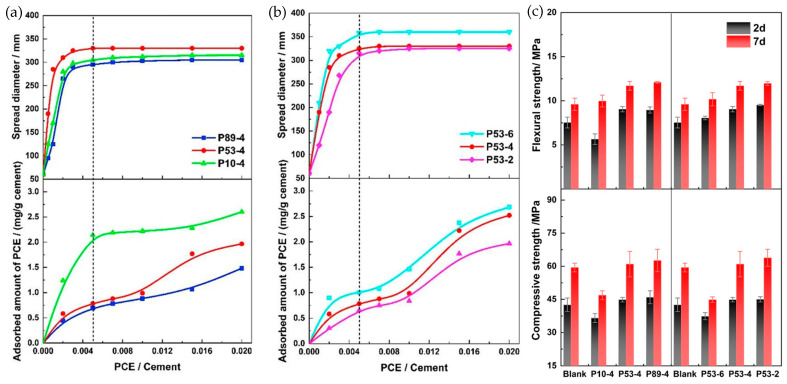
Effects of various types of PCE dosages on mortar fluidity, adsorption, and mechanical properties. (**a**) Effects of different side chain lengths on fluidity and adsorption; (**b**) effects of varying side chain densities on fluidity and adsorption; (**c**) effects of different types of PCEs on mechanical properties. Adapted from [62] (permission has been granted).

Zhang et al. [62] synthesized polycarboxylate ethers (PCEs) using acrylic acid (AA) and α-methylallyl-ω-hydroxypolyethylene glycol ether (HPEG) as primary monomers. By employing HPEG macromonomers with 53 repeating units (−CH_2_−CH_2_−O−), PCEs with AA/HPEG ratios of 6:1, 4:1, and 2:1 were prepared. The resulting polymers were designated as P53-6, P53-4, and P53-2, corresponding to their side chain density from high to low. Additionally, using HPEG macromonomers with 89, 53, and 10 repeating units (−CH_2_−CH_2_−O−), while maintaining the AA-to-HPEG ratio at 4:1, PCEs with varying side chain lengths were synthesized. These polymers were labeled P89-4, P53-4, and P10-4, reflecting their side chain lengths from high to low. The chemical structure of the synthesized PCEs is illustrated in Figure 2. The incorporation of different types of PCEs enhanced the fluidity of the mortar. When the side chain density was held constant, PCEs with shorter side chain lengths (Figure 3a) and those with a lower side chain density (Figure 3b) for the same side chain length exhibited higher adsorption capacity. Furthermore, it was observed that PCEs with longer side chains and higher side chain density were more favorable for early strength development (Figure 3c). In summary, as the side chain length and density of the water reducer increase, the adsorption amount decreases, while the early strength of the mortar increases [37,62,67,69,70].

**Figure 4:**
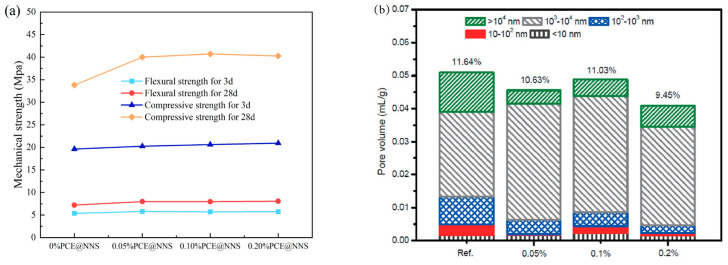
Effects of different PCE@NNS dosages on mechanical properties and porosity of mortar: (**a**) mechanical properties and (**b**) pore distribution. Adapted from [72] (permission has been granted).

Nanotechnology is an emerging field that manipulates and controls matter at the molecular level. It has enabled the scientific community to develop a variety of advanced materials applicable across multiple disciplines [71]. Miao et al. [72] utilized colloidal nano-silica (NNS) to synthesize polycarboxylate ether (PCE) containing Si-OH functional groups. Due to its superior mechanical strength, ultrafine size, pozzolanic reactivity, and excellent compatibility with cement hydrates, this new type of PCE can enhance the mechanical properties (Figure 4a) and reduce porosity (Figure 4b) of mortar while maintaining good dispersibility. The aforementioned studies indicate that modifying the structure of PCE polymers can significantly improve fluidization efficiency [73,74] and the retardation effect of PCE [75,76,77,78].

#### 3.4.2. Water Retention Agent

**Figure 5:**
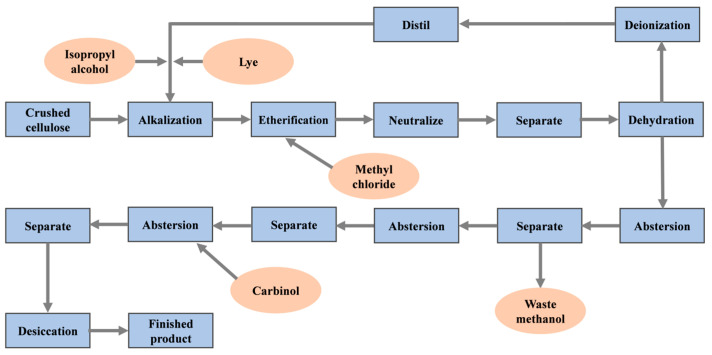
Typical manufacturing process of cellulose ether.

Water-retaining agents primarily consist of water-soluble polymer compounds, including polyacrylates, cellulose, and natural rubber. Cellulose ether (CE) is a widely utilized water-soluble polymer for water retention modification in CSL, owing to its exceptional water-retaining and thickening properties [13,69,79,80]. CE includes hydroxypropyl methyl cellulose (HPMC), hydroxyethyl methyl cellulose (HEMC), and hydroxyethyl cellulose (HEC) [69,81]. In the production process, CE is reactivated using active sodium and subsequently reacted with functional polymers, such as methyl chloride, ethylene oxide, or propylene oxide, to produce various types of cellulose ethers [69]. The typical manufacturing process of cellulose ether is illustrated in Figure 5.

**Figure 6:**
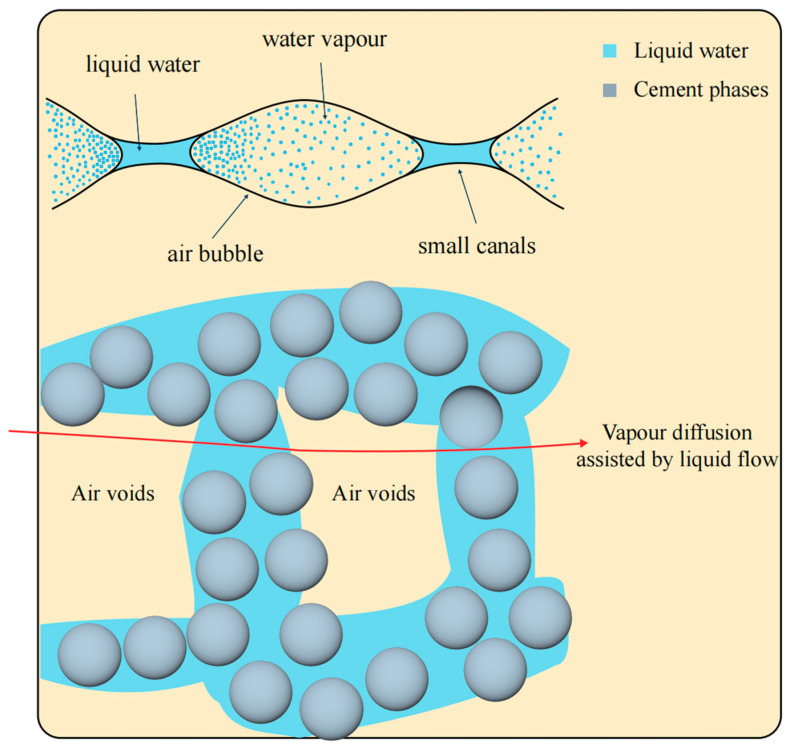
Modeling of the porous network characteristics of HPMC, and describing water transport through this specific microstructure [81].

**Figure 7:**
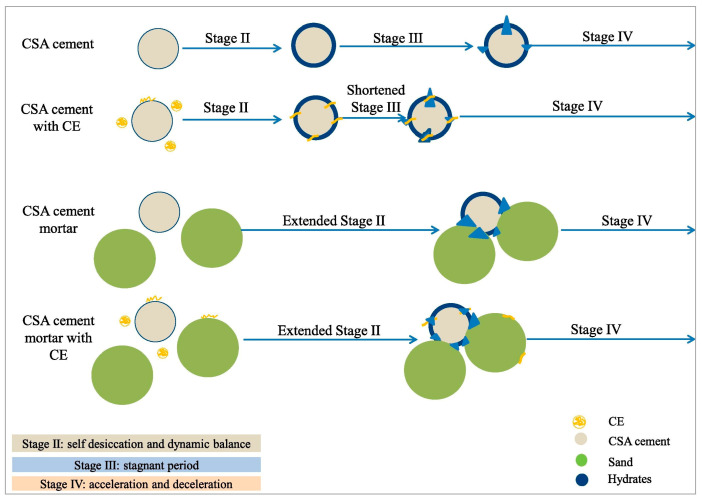
Effect of CE on the hydration process of CSA cement mortar [82] (permission has been granted). The yellow line in the diagram indicates the CE molecules embedded on the surface of the cement particles, and the blue triangle indicates the accumulated hydration products. These figures are the different characteristics of CE and Hydrates in the image annotation information.

**Figure 8:**
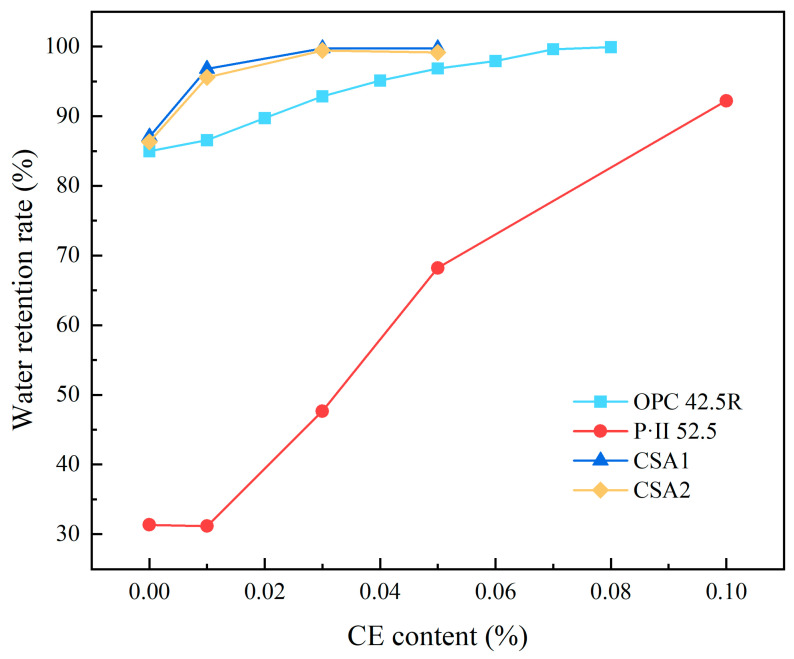
Effect of CE on the water retention of different types of cement (among these, OPC refers to ordinary Portland cement, P·II denotes Portland cement with an addition of 5% limestone or slag, CSA2 contains a higher proportion of dicalcium silicate (C_2_S), and CSA1 has a greater amount of anhydrous calcium sulphoaluminate).

Pourchez et al. [81] found that CE is a significant controlling factor for water transport and the evolution of porous structures in mortar. The addition of CE can increase the number of pores and capillaries within the mortar. A reduction in pore size slows the transfer rate of fluid in the liquid phase. Vapor diffusion, assisted by liquid flow, primarily occurs in larger voids (Figure 6), thereby enhancing the water retention capacity of the mortar. Li et al. [82] proposed a model, illustrated in Figure 7, to explain how CE functions as a medium for water migration, influencing the hydration of CSA cement/mortar under various conditions. A CE film forms within the cement matrix. As a hydrophilic material, this CE film likely serves as a conduit for water to penetrate partially hydrated cement particles, thereby reducing the duration of the stagnation period. In CSA mortar containing sand fillers, the sand is embedded within the cement hydrate. This can lead to the formation of defects or channels at the interface between the cement hydration products and the sand, which allows for water to migrate to the non-hydrated cement particles. This process results in slow but continuous hydration, making it challenging for the mortar hydration to reach a stagnation period. The addition of a chemical additive, such as a water-retaining agent, regulates the migration rate of water within the cement/mortar matrix and promotes the hydration reaction. Patural et al. [15] found that water-retaining agents can enhance the consistency of fresh mortar, in addition to improving water retention, workability, and the usable time of the mortar. The dosage of CE significantly influences the water retention mechanism of CSL. At low doses, water retention is achieved through intramolecular water adsorption and associated swelling. At higher doses, a hydrogel-like three-dimensional polymer network forms, which blocks pores in the mortar matrix, thereby enhancing water retention [83,84,85]. As the molecular weight of CE increases, the yield stress decreases, consistency improves, and water retention increases. A comparative study examining the impact of CE on the water retention properties of various cement mortars (Figure 8) found that even a low content of CE can significantly enhance the water retention properties of different types of mortars [86,87,88].

**Figure 9:**
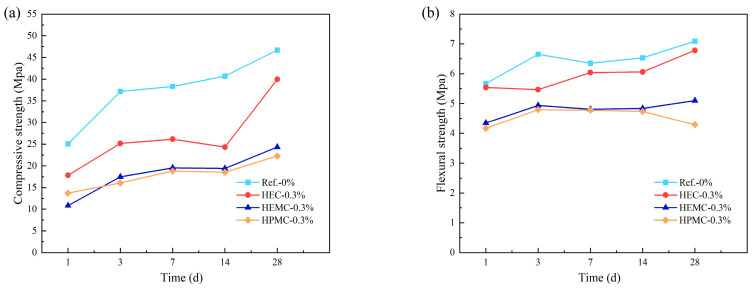
Effects of different types of CE on the mechanical properties of mortar: (**a**) compressive strength and (**b**) flexural strength.

The influence of CE on mortar is also evident in its mechanical properties. Kim and Kang [89] found that the addition of CE increased the water retention of the mortar, which facilitated the hydration of C_3_S and C_2_S, thereby improving the bonding strength of the mortar after drying. Li et al. [69] investigated the effects of three types of CE—HEC, HEMC, and HPMC—on the mechanical strength of CSA cement mortar (Figure 9). They discovered that HEC had the least impact on the mechanical properties of the mortar. However, CE also delays the hydration of cement, which prolongs the setting time and reduces early strength. These effects are generally recognized as the side effects of CE [90,91,92].

#### 3.4.3. Retarder

Unlike the rheological optimization provided by PCE or the moisture retention offered by CE, set retarders extend the setting time by slowing down the hydration reaction rate. This extension ensures adequate working time for large-scale construction projects while preventing plastic cracking that can occur due to rapid water evaporation [93,94,95]. Commonly used retarders include tartaric acid (TA) [96,97], citric acid (CA) [98,99,100], sodium tripolyphosphate (STPP) [101,102] and protein salt (PS) [2]. These retarders are typically low-molecular-weight organic compounds that contain multiple carboxyl groups. The low molecular weight characteristic facilitates the adsorption of acid radical ions onto the surface of Portland cement particles, thereby delaying cement hydration [103]. STPP is classified as an alkaline phosphate retarder. These alkaline phosphate retarders exist in the form of sodium salts and contain multiple identical [NaPO_3_]^2−^ structural units (Table 2), which contribute to their excellent retarding performance [104]. Proteins, characterized by their green, renewable, abundant, and non-polluting nature, have emerged as potential retarders for the CSL industry and have been extensively studied [104,105,106].

**Figure 10:**
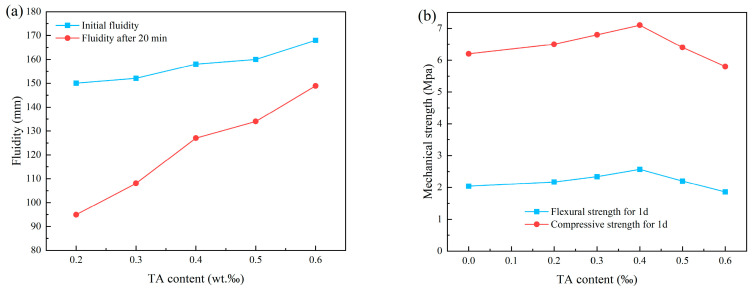
Effect of different TA dosages on mortar properties: (**a**) fluidity and (**b**) mechanical properties.

**Figure 11:**
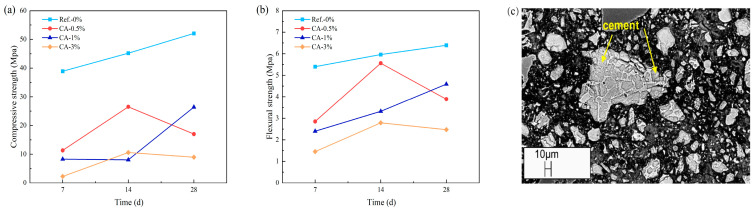
(**a**) Effect of different CA dosages on mortar compressive strength; (**b**) effect of different CA dosages on mortar flexural strength; (**c**) SEM image of mortar with 3% CA dosage at 28 days. Adapted from [103] (permission has been granted).

TA is a green retarder that can be produced from waste materials in the wine or juice industry [107,108]. Wang et al. [13] investigated the effects of adding TA on the fluidity (Figure 10a) and mechanical properties (Figure 10b) of CSL. The addition of TA slowed down the hydration reaction process of the mortar, prolonged the setting time, and increased the fluidity of CSL within the same time frame. The mechanical properties initially increased and then decreased with the rising TA content, peaking at a TA concentration of 0.4 wt‰. Notably, after 5 h, the blocking effect of TA on silicate minerals diminished, which facilitated an increase in the precipitation rate of hydration products such as calcium hydroxide and helped maintain the early strength of the mortar [109]. Kastiukas et al. [103] studied the effect of CA on the early engineering properties of CSL. Their findings indicated that the incorporation of CA resulted in a reduction of both compressive strength (Figure 11a) and flexural strength (Figure 11b) of the mortar. Furthermore, a higher concentration of CA inhibited the dissolution of cement particles (Figure 11c). J. Plank elucidated the incompatibility between cement and CA in CSL through the lens of competitive adsorption. CA demonstrates a stronger competitive adsorption capability compared to tartaric acid (TA), leading to a more pronounced detrimental effect on the mechanical properties of CSL [110]. Ltifi et al. [111] conducted various tests on PC with different amounts of STPP and found that as the STPP content increased, both the viscosity of the mortar and the degree of delay in cement hydration also increased. This phenomenon occurs because STPP reacts with Ca^2+^ ions in the cement mortar to form the complex [CaP_3_O_10_]^3−^. This complex subsequently interacts with Ca^2+^ and Al^3+^ ions on the surface of cement particles, creating an electric double-layer structure that enhances electrostatic repulsion between the particles. Consequently, the contact area between cement particles and water is reduced, delaying the cement hydration process and thereby achieving a retarding effect [112].

**Figure 12:**
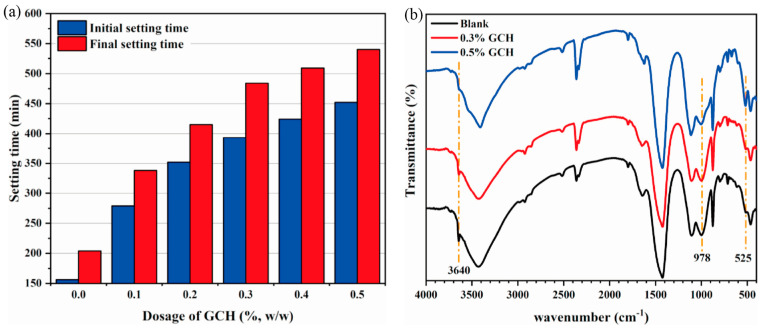
(**a**) The relationship between the amount of GCH and the setting time of cement; (**b**) FTIR spectra of cement mortar with varying GCH content [106] (permission has been granted).

**Figure 13:**
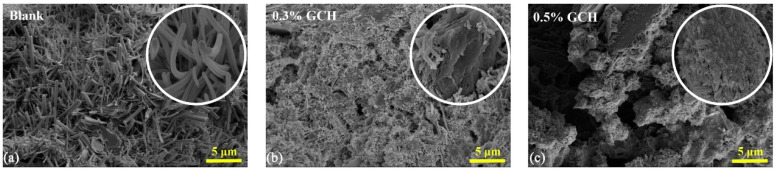
The effect of varying GCH contents on the microstructure of hardened cement: (**a**) 0%, (**b**) 0.3%, (**c**) 0.5% [106] (permission has been granted).

Ding et al. [106] utilized acrylic acid (AA) and acrylamide as raw materials to modify collagen hydrolysate (CH) derived from chrome waste through graft copolymerization, resulting in the synthesis of grafted collagen hydrolysate (GCH) for cement retardation. As the dosage of GCH increased, the setting time of the cement also extended (Figure 12a), indicating that GCH exhibits a significant cement retardation effect. Figure 12b illustrates that the addition of GCH inhibits the formation of Ca(OH)_2_ at 3640 cm^−1^ in the infrared spectrum, with this inhibitory effect becoming more pronounced as the amount of GCH increases. Figure 13a shows that cement without GCH consists of uniform needle-shaped crystals that are intertwined. In contrast, the incorporation of GCH leads to a reduction in mesh-like crystals, resulting in a smoother cement structure (Figure 13b,c). This study demonstrates that GCH not only influences the hydration rate of cement reactants, but also alters the crystal morphology of the hydration products. Their research not only effectively recovers chrome scale waste, but also provides a valuable reference for the development of protein-based retarders in CSL.

#### 3.4.4. Other Admixtures

In addition, redispersible latex powder [113], defoaming agent [114], suspension stabilizer [115], slump retaining agent [46,116], expansion agent [117], and accelerating admixture [118] are also used in combination with CSL. The incorporation of these admixtures can reduce the porosity of CSL materials, enhance the initial hydration rate, prevent mortar segregation, and minimize shrinkage in the mortar. However, there is limited research on these admixtures, and further studies are necessary to elucidate the mechanisms and assess the feasibility of these admixtures in influencing the performance of CSL.

## 4. New Green CSL

### 4.1. Recycling Industrial Waste and By-Products During CSL Production

**Figure 14:**
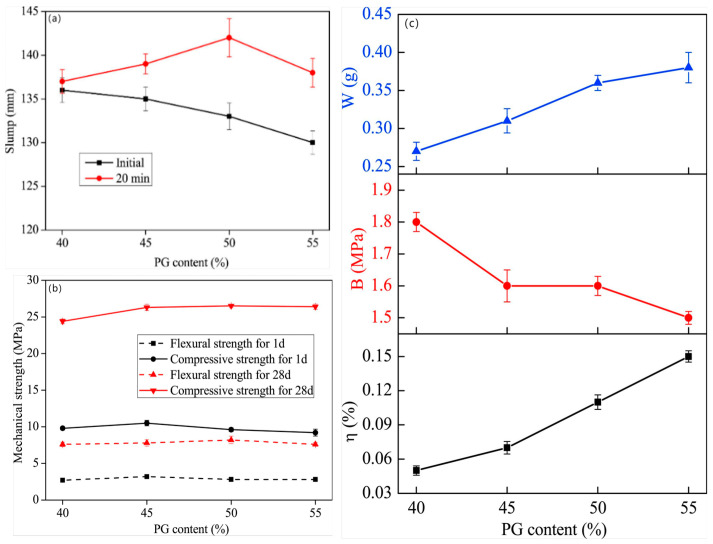
(**a**) Effect of PG content on the fluidity of CSL; (**b**) effect of PG content on the flexural and compressive strength; (**c**) effects of PG content on shrinkage (η), bond strength (B) and wear resistance (W) [5] (permission has been granted).

**Figure 15:**
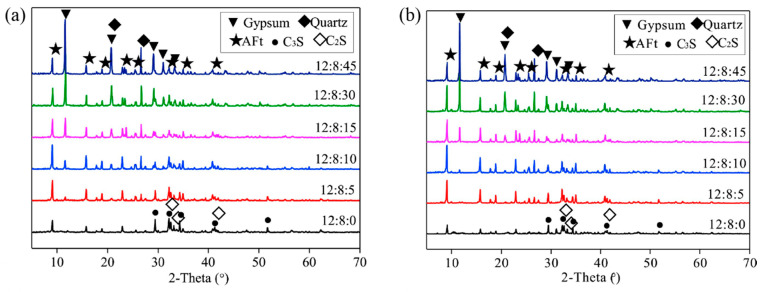
XRD patterns of hardened pastes with different PG content: (**a**) 1 day, (**b**) 28 days [5] (permission has been granted).

The production of cement in CSL will lead to an increase in greenhouse gas emissions, including CO_2_, NO_x_, and SO_x_ [38]. However, certain by-products, such as by-product gypsum, limestone powder (LP), and porcelain waste, can serve as substitutes for natural resources and cement in the composition of CSL. The reuse of these industrial by-products not only reduces the energy consumption associated with processing natural resources, but also contributes to a decrease in greenhouse gas emissions and other harmful pollutants [119]. Lin et al. [5] explored the use of raw by-PG as a material for producing CSL. For CSL mixed with PG, it is essential to achieve good flowability, mechanical properties, bonding strength, and wear resistance. Research has demonstrated that CSLs containing more than 40% PG exhibit improved fluidity (Figure 14a) and do not experience significant loss of mechanical properties (Figure 14b). This occurs because when PCE is incorporated into the system with gypsum particles, the gypsum particles rapidly release Ca^2+^ and SO_4_^2−^ into the solution, exposing a large number of active sites. The carboxyl groups in PCE molecules react with Ca^2+^ to form compounds that adsorb onto the surface of the gypsum particles, providing steric hindrance that facilitates the dispersion of the gypsum particles and enhances their mobility [120]. In addition, as the content of PG increases, the shrinkage rate, bonding strength, and wear resistance of CSL all decrease (Figure 14c). According to the performance requirements outlined in JC/T 985-2017 [1], the PG content should not exceed 55%. Furthermore, to investigate the role of PG in CSL, X-ray diffraction (XRD) analysis was conducted on CSL samples with varying levels of PG doping. Figure 15 presents the XRD pattern of the hardened slurry with a composition of CSA: OPC: PG = 12: 8: x. It was observed that the volume of AFt increased with rising PG content and stabilized when x > 10. When x ≤ 10, the diffraction peak of gypsum was detectable at 1 day (Figure 15a). As hydration progressed, the diffraction peak of gypsum disappeared by 28 days (Figure 15b), indicating that the PG in CSL could be completely consumed. However, when x > 10, an excessive amount of PG may lead to a decline in CSL performance.

**Figure 16:**
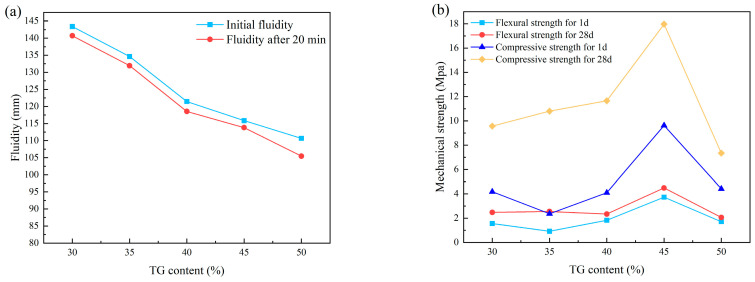
(**a**) The effect of TG content on the fluidity of CSL; (**b**) effect of TG content on the mechanical properties of CSL.

**Figure 17:**
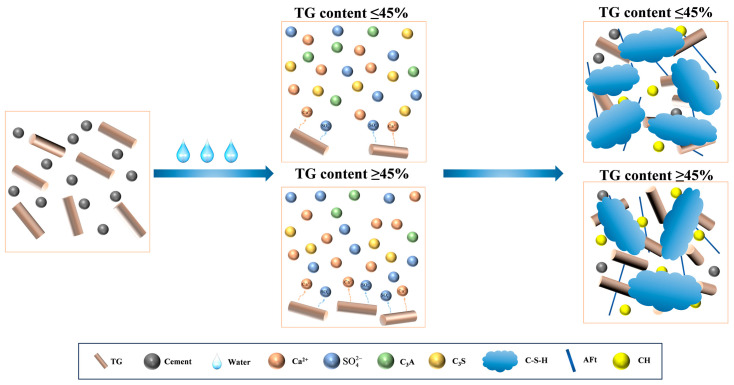
The influence mechanism of different TG contents on the hydration process of CSL.

Titanium gypsum (TG) is an industrial solid waste primarily composed of CaSO_4_·2H_2_O. Due to its high free water content, significant impurity levels, poor mechanical properties, and challenges associated with recycling, the annual emissions of TG in China have reached 30 million tons [121]. Currently, research has explored the use of TG as a cement retarder, a binding material, and for the production of lightweight wall materials and roadbed materials; however, the application of TG remains limited [122,123,124,125]. Wang et al. [23] utilized TG as a raw material for the preparation of CSL, incorporating varying proportions of TG to replace cement in CSL. Research has demonstrated that, under a fixed PC/CSA ratio, the flowability of the mortar decreases as the amount of TG increases (Figure 16a). This trend can be attributed to the microstructure of TG, which predominantly consists of long columns and flakes, along with a significant quantity of spherical and flocculent impurities. The irregular accumulation of minerals in diverse shapes creates additional pores, leading to increased water absorption and reduced fluidity of the mortar. After the addition of TG to the CSA-PC-based mortar, the C-S-H gel produced by cement hydration fills the spaces between the TG structures, enhancing the compactness and mechanical properties of the mortar. However, an excessively high TG ratio can diminish the amount of C-S-H gel generated during cement hydration, leading to a reduction in strength (Figure 16b). Furthermore, under the influence of the C-S-H gel, some TG participates in the hydration process of C_3_A, resulting in the formation of additional Aft. Conversely, when the TG content is too high, the C-S-H gel is compromised by an excess of CaSO_4_·2H_2_O. In this scenario, TG primarily acts as a filler within the material, which negatively impacts the microstructure and compactness of the cement-based matrix. The underlying mechanism is illustrated in Figure 17. Additionally, the Ca^2+^ ions released by TG chelate with the retarder in the CSL system, leading to a reduction in both Ca^2+^ concentration and ionic activity. This decrease, in turn, inhibits the hydration of gypsum crystals as they transition from the hemihydrate to the dihydrate form [105].

Pereira and Camarini [126] investigated the feasibility and applicability of incorporating two types of porcelain waste into CSL, using porcelain waste as a substitute for cement. They concluded that although the two types of porcelain waste reduced flow and prolonged the setting time, they enhanced the compressive strength of CSL. Additionally, the water absorption and permeability of CSL mixed with porcelain slag met the specifications’ requirements.

The commonalities identified in the studies above suggest that it is feasible to partially substitute CSL materials with industrial by-products in CSL production. By carefully controlling the proportion of industrial by-products incorporated, the overall properties—such as mechanical strength, flowability, setting time, bonding strength, shrinkage rate, and wear resistance—of CSL containing these by-products can effectively meet the requirements outlined in the Chinese standard JC/T 985-2017 for CSL performance.

### 4.2. Historical Research on and Latest Engineering Applications of Industrial By-Products in CSL Production

Table 3 summarizes the application dosage and performance impact of various industrial by-products in CSL in chronological order. The data indicate that most industrial by-products, or their combinations, such as plastic waste (PW), white ceramic waste (CB), natural phosphogypsum (PN), red ceramic waste (C), insulator porcelain waste (P), PG, phosphorus building gypsum (PBG), flue gas desulfurization gypsum (FGDG), natural anhydrite (NA), ground slate (GS), and sugarcane bagasse ash (SCBA), have varying effects on CSL mixtures. These industrial by-products influence the performance of CSL in both fresh and hardened states in different ways.

The summary table indicates that incorporating white ceramics into CSL results in a lower shrinkage rate and enhanced mechanical properties [26]. One of the most prevalent industrial by-products, PG, can significantly improve the flowability of CSL due to the smaller particle size of PG waste after calcination, which enhances its flow characteristics [26]. However, adding more than 55% PG may lead to a decline in the performance of CSL mixtures, potentially due to variations in gypsum hydration products and bonding structures [21]. When these green adhesives are added individually, PG demonstrates the most significant improvement in processability; however, as the amount of PG increases, the early strength of the mixture tends to decrease. An analysis of the flowability and hardening properties of CSL reveals that PG is a promising candidate material for CSL applications [21,26,126,127]. Additionally, some studies have highlighted the effects of other industrial by-products, such as NA, SCBA, GS, and FGDG, on the performance of CSL [11,128,129,130].

**Table 3:** Basic research on the preparation of CSL using different green materials.

Year	Green Binder	Other Material	Dosage	Mortar Property									Ref.
				A	B	C	D	E	F	G	H	I	
2010	GS	PC, Sand	GS: 0–45 total wt. %	√		X	X	X	X			√	[129]
2013	PW	PC, Sand, LP	PW: 0–50 total wt. %	√		X	X		√		√		[27]
2016	PG	PC, CSA, Sand	PG: 45–55 total wt. %			√	√	X		X			[5]
2017	PG	PC, CSA, Sand	PG: 19.80 total wt. %	√	√	X			X		X		[6]
2017	NA	CSA, Sand, LP	NA: 0–35 total wt. %	√	√	√	√	√					[11]
2018	P, C	PC, Sand	P: 15–50 PC wt. %			√	√				√	X	[126]
2018	P, C	PC, Sand	C: 15–50 PC wt. %			√	√				X	X	[126]
2019	PBG	CSA, PC, Granulated blast furnace slags, Sand	PBG: 50 total wt. %	√	√	√	√	√		√			[127]
2020	Marble and granite cutting waste (MGCW)	PC, Sand	MGCW: 40–50 total wt. %	√	√	√	√		√		√	√	[131]
2020	SCBA	PC, Sand, LP	SCBA: 0–30 total wt. %	X		√	√		√	√	X		[128]
2021	FGDG	PC, Mechanochemical Syngenite, Sand, Quartz flour	FGDG: 35 total wt. %	X	X	√	√	√	√				[130]
2023	fluorogypsum (FLG)	PC, Sand	FLG: 92 total wt. %	√	√	√	√		√	√			[19]
2024	FGDG, PG	CSA, Sand	PG: 90 total wt. %	√	√	√	√						[21]
2024	FGDG, PG	CSA, Sand	FGDG: 90–100 total wt. %	√	√	√	√						[21]
2024	Red mud (RM)	PG, FA, Sand, Quicklime, PC	RM: 11–26 total wt. %	√	√	√	√						[132]
2024	PG	RM, FA, Sand, Quicklime, PC	PG: 11–26 total wt. %	√	√	X	X						[132]
2024	PG, CB, PN	PC, Sand	PG: 474.09 kg/m3	√	√	X	√	X	√				[26]
2024	PG, CB, PN	PC, Sand	CB: 474.09 kg/m3	√	√	√	√	√	√				[26]
2024	PG, CB, PN	PC, Sand	PN: 474.09 kg/m3	√	X	X	√	X	√				[26]

Note: A: liquidity; B: setting time; C: compressive strength; D: flexural strength; E: shrinkage rate; F: porosity; G: slump; H: water absorption; I: segregation. The symbols √ and X represent the positive and negative effects of industrial by-products on the performance of CSL, respectively.

## 5. Conclusions

This study presents the testing indicators and performance requirements for CSL in international and Chinese standards. It reviews the composition materials of CSL, discusses the types of additives used in CSL, and examines their impact on CSL performance. Additionally, the role and effectiveness of industrial by-products applied in CSL were thoroughly explored and compared. The following conclusions were drawn:

1. The rapid popularization of CSL is directly related to the standardization framework established by current standards (such as JC/T 985, ISO). These standards provide a clear benchmark for the normative application of CSL through quantitative indicators.
2. The performance of CSL is mainly affected by the composition of cementitious materials, aggregates, and fillers. Through the performance analysis of different CSLs, it can be seen that the addition of CSA instead of PC can optimize the formation of ettringite, thereby increasing the compressive strength of 28 days and reducing carbon dioxide emissions by 30% to 50%. The fine sand with a particle size of 0–1 mm has the best performance in CSL. Fly ash can enhance the fluidity and stability of CSL and improve its compactness and mechanical properties.
3. Water reducers, water retainers, and retarders significantly enhance the fluidity, mechanical properties, and durability of CSL by optimizing the water–cement ratio, regulating the hydration process, and improving the microstructure. Among these additives, the molecular structure of PCE (side chain length and density) directly influences its adsorption characteristics and early strength development; cellulose ether improves water retention by forming a three-dimensional network; however, excessive use of these admixtures may delay hydration and diminish early strength.
4. Industrial by-products such as PG, TG, ceramic waste, and limestone powder can partially replace traditional cementitious materials or aggregates, thereby reducing the environmental impact of CSL. When PG is added to the CSL system at a concentration of 55% or less, the performance of the mortar can be improved, and carbon emissions can be reduced. The incorporation of ceramic waste at 55% can enhance compressive strength, reduce shrinkage, and prolong setting time. By controlling the incorporation ratio of industrial by-products, various green CSLs can meet the requirements of mechanical properties and workability in relevant standards (JC/T 985-2017).
5. The use of a single admixture or by-product presents certain limitations, including the negative impact of PCE on early strength and the interference of impurities found in industrial waste with workability. To enhance overall performance, it is essential to utilize combinations of multiple admixtures or to implement synergistic modifications using by-products, such as a composite system that incorporates both fly ash and limestone powder. Additionally, the environmental risks related to the toxicity and heavy metal leaching of industrial by-products necessitate a systematic evaluation.

## 6. Outlook and Prospect

CSL has a wide range of applications across various fields of civil engineering. Based on the observations presented in this review, the author proposes several aspects that should be considered in future CSL research:

1. Future aims should be to advance the research and development of high-durability CSL materials that incorporate integrated functionalities, including waterproofing, fire resistance, and antimicrobial properties, as well as establish a comprehensive lifecycle performance evaluation system that encompasses flatness retention rate, crack resistance coefficient, and color difference stability, while ensuring the functional stability of the materials under extreme temperature and humidity conditions.
2. In subsequent research, fiber materials and nano-mineral additives can be incorporated into mortar to enhance its strength, toughness, and durability. Simultaneously, the balance between compressive strength and initial fluidity was optimized through orthogonal testing.
3. While evaluating the performance of admixtures, it is essential to consider their potential negative effects on CSL properties. Addressing the issue of crystal structure alteration caused by admixtures represents a crucial direction for the future advancement of CSL technology. Furthermore, the development of new admixtures must also fully leverage the unique chemical structures and mechanisms of action of existing admixtures.
4. There are still many areas that require further investigation regarding the influence of gypsum on the performance of CSL. Future research will focus on conducting pre-treatments, such as dehydration, alkalization, ball milling, and calcination, on various industrial by-product gypsum to enhance the compatibility between gypsum crystals and cement paste.
5. Various combinations of industrial by-products can be utilized to produce CSL, leveraging the synergistic effects of these by-products to enhance both the fresh and hardening performance of CSL. Materials exhibiting performance deficiencies can be compensated for by incorporating alternative materials. Furthermore, it is essential to explore the potential of other environmentally friendly adhesives in CSL, as many remain underutilized and have not been fully developed. This category of industrial waste and by-products includes polymer resins, pollutants from tannery wastewater, ash from Prosopis juliflora, thermosetting plastic waste, waste glass, eggshells, sludge from sewage treatment plants, ground concrete waste powder, and undeveloped waste from the paper and pulp industry, among others.
6. When utilizing industrial by-products, it is essential not only to consider the properties of the materials themselves but also to assess their environmental impact, including factors such as toxicity, corrosiveness, leaching rates of heavy metals, flammability, and reactivity. The integration of mass spectrometry with gas chromatography or liquid chromatography is facilitated by a comprehensive four-stage evaluation framework. This framework includes screening, mechanism analysis, process simulation, and engineering validation, enabling full-chain risk management of industrial by-products from laboratory research to engineering applications.

## Figures and Tables

**Table 1:** CSL characteristics, and the standards and test requirements.

CSL Property	Standard Test	Description	Requirement (JC/T 985-2017) [1]
Appearance	JC/T 985-2017 [1]	Cementitious self-leveling compound for floor	Uniform, no lumps, no other objects
Initial fluidity/mm	ISO 9597:2008 [28]	Cement—Test methods—Determination of setting time and soundness	≥130
20 min fluidity/mm			≥130
24 h flexural strength/MPa	ISO 679:2009 [29]	Cement—Test methods—Determination of strength	≥2.0
24 h compressive strength/MPa			≥6.0
28 d flexural strength/MPa			≥6.0
28 d compressive strength/MPa			≥25.0
28 d tensile bond strength/MPa	ISO 13007-2:2013 [30]	Ceramic tiles—Grouts and adhesives—Part 2: Test methods for adhesives	≥1.5
Shrinkage/%	JGJ/T 70-2009 [32]	Standard for test method of performance on building mortar	−0.10~+0.10
Impact resistance	JC/T 985-2017 [1]	Cementitious self-leveling compound for floor	No cracking or detachment from the base plate
Wear resistance/mm^3^	ISO 10545-6:2010 [31]	Ceramic tiles-Part 6: Determination of resistance to deep abrasion for unglazed tiles	≤400

**Table 2:** Performance studies of some retarders.

Organic Acid Retarder	Structural Structure	Functional Group	Molecular Weight
Citric acid (CA)	** 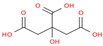 **	—COOH —OH	192
Tartaric acid (TA)	** 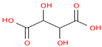 **	—COOH —OH	150
Sodium tripolyphosphate (STPP)	** 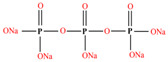 **	—	368

## Data Availability

No new data were created or analyzed in this study.
